# Immunohistochemical Profile of a Conspicuously Organized Structure in the Dorsal Forebrain of the Peacock Gudgeon, *Tateurndina ocellicauda*

**DOI:** 10.1002/cne.70097

**Published:** 2025-10-15

**Authors:** Ruth Gutjahr, Maximilian S. Bothe, Boris P. Chagnaud

**Affiliations:** ^1^ Department of Biology University of Graz Graz Austria

**Keywords:** gobies, immunohistochemistry, neuronal layers, pallium, teleost

## Abstract

In the pallium of mammals and reptiles, layered neuronal organizations appear as a key feature to facilitate neuronal processing. Such layered organizations allow to segregate inputs and information processing into different neuronal compartments and are used in a variety of tasks, including multisensory integration and the formation/retrieval of memories. Although teleost fishes also need to process multisensory information and form memories, their pallium generally does not contain any layered structures. Instead, neurons appear to be organized into nuclei with loosely arranged cells. One exception to this general principle can be found in gobiiform fishes: Their caudo‐dorsal telencephalon is, in part, marked by neurons, which are organized into several soma‐dense and fiber‐rich subregions, which are referred to as Dl4–Dl7 in lateral to medial order. To better understand the organization of this structure, we investigated the immunoreactivity of these subregions using a variety of different antigen targets: tyrosine hydroxylase (TH), parvalbumin (PV), substance P (SP), calretinin (calret), neurofilament heavy chain (NFH), choline acetyltransferase (ChAT), and gamma‐aminobutyric acid (GABA). Our results reveal a differential distribution of the investigated antigens throughout the subregions, with immunoreactivity hotspots of dense putative terminal fields in different fiber‐rich subregions, depending on the antigen investigated. Within Dl7, dense fiber fields were most immunoreactive for calret and PV, within Dl6 for PV, SP, and NFH, within Dl5 for ChAT, and within Dl4 for calret. This differential distribution shows that individual subregions receive input from different sources, which, in turn, suggests that they are different functional compartments.

## Graphical abstract

In the dorsal telencephalon of gobiiform fishes, there is a conspicuously organized structure, consisting of readily identifiable subregions that are surrounded by layers of cell bodies. Using immunohistochemistry against several antigen targets in this structure, we demonstrate that each of these subregions is characterized by its own individual neurochemical profile, which indicates corresponding differential inputs into different subregions.

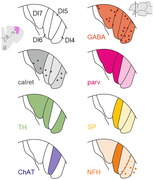

**Abbreviations**
calret: calretinin
ChAT: choline acetyltransferase
Dc: central zone of the dorsal telencephalon
Dd: dorsal zone of the dorsal telencephalon
Ddi: intermediate region of the dorsal zone of the dorsal telencephalon
dDl: dorsal zone of Dl
dDm: dorsal zone of Dm
Dl: lateral zone of the dorsal telencephalon
Dl1‐3: subregions 1–3 of the dorsolateral telencephalon
Dl4‐7: subregions 4–7 of Dl
Dm: medial zone of the dorsal telencephalon
Dp: posterior zone of the dorsal telencephalon
dVd: dorsal zone of Vd
Dx: zone “X” of the dorsal telencephalon
EN: entopeduncular nucleus
GABA: gamma‐aminobutyric acid
ir: immunoreactive
L:D cycle: light:dark cycle
lfb: lateral forebrain bundle
MS‐222: tricaine methanesulfonate 222
n.a.: not available
NFH: neurofilament heavy chain
o.n.: overnight
parv: parvalbumin
PB: phosphate buffer
PBS: phosphate buffered saline
PG: preglomerular complex
PG1: nucleus 1 of the preglomerular complex
PG2: nucleus 2 of the preglomerular complex
POA: preoptic area
PTh: prethalamic nucleus
PTh‐l: lateral prethalamic nucleus
PTh‐m: medial prethalamic nucleus
PV: parvalbumin
SP: substance P
TE: telencephalon
TeO: optic tectum
TH: tyrosine hydroxylase
V: ventral telencephalon
Vc1: region 1 of the central zone of the ventral telencephalon
Vd: dorsal zone of the ventral telencephalon
vDl: ventral zone of the Dl
Vl: lateral zone of the ventral telencephalon
Vv: ventral zone of the ventral telencephalon
vVd: ventral zone of Vd

## 1 Introduction

The mammalian pallium is well‐known for its outstanding processing abilities, such as the integration of multisensory information (Ghazanfar and Schroeder 2006; Meijer et al. 2019; Sepulcre et al. 2012), its involvement in the formation and recalling of memories (Broadbent et al. 2004; Mishkin 1978; Scoville and Milner 1957; Squire and Wixted 2011), and its contribution to spatial navigation (Moser et al. 2017; O'Keefe 1976; O'Keefe and Dostrovsky 1971; O'Keefe and Nadel 1978). A chief underlying foundation of these processes resides in highly organized, layered structures, such as the six‐layered isocortex (Hubel and Wiesel 1965, 1972) and the three‐layered hippocampus (Broadbent et al. 2004; O'Keefe 1976).

The need for processing diverse sensory input and recalling certain locations is, however, not unique to mammals. Consequently, layered pallial structures also appear across some nonmammalian vertebrates: Avian (Güntürkün 2012; Stacho et al. 2020) and non‐avian (de Carvalho Pimentel et al. 2011; Reiner 1993) reptiles possess cortex‐like structures with differing numbers of layers (Naumann and Laurent 2020) in contrast to the six layers in the mammalian isocortex. In the area dorsalis of teleosts, which corresponds to the mammalian pallium (Kage et al. 2004; Wullimann and Mueller 2004), a loose organization of neurons into separate regions can be differentiated (Nieuwenhuys 1963, 1982; Wullimann et al. 1996). None of these regions, that is, the central (Dc), the medial (Dm), the lateral (Dl), the dorsal (Dd), and the posterior (Dp) parts (Meek and Nieuwenhuys 1998; Nieuwenhuys 1963), are known to be organized in layers. However, evidence of some hidden Dl‐layering has been reported (Trinh et al. 2016).

One prominent exception to this loose organization of neurons in the teleostean pallium is present in the dorsal part of the telencephalon of gobiiform fishes (Gebhardt 2022; Hagio et al. 2021, 2018; Jiang et al. 2023; Kawaguchi et al. 2019; Kusunoki et al. 1977; Yoshida et al. 2020). Here, neurons are arranged into well‐defined soma‐dense layer‐like subregions that are separated by fiber‐rich subregions. In the yellowfin goby *Rhinogobius flumineus*, for example, Dl has been described as consisting of seven different subregions (Dl1–Dl7), which were identified using histochemistry, as well as tracer injections into the prethalamic nucleus (PTh) (Hagio et al. 2021, 2018), which belongs to the preglomerular complex (Meek and Nieuwenhuys 1998). Four of these seven subregions (Dl4–Dl7 in lateral to medial order) are organized in the above described “layered” fashion and have collectively been termed “Dx” (Gebhardt 2022). Although in four of six sampled gobiiform families (there are eight in total (Nelson et al. 2016)), a Dx has been identified (Gebhardt 2022), the anatomical organization of the different Dx‐subregions is subject to diversity. In the peacock gudgeon, *Tateurndina ocellicauda* (a member of the eleotrid family), for example, Dl7–Dl5 of Dx can be readily detected (Gebhardt 2022), whereas Dl4 is more difficult to discern.

Recent tracer injections have revealed sources of ascending information to different forebrain areas of the peacock gudgeon, including Dx (Gutjahr et al. 2025). Within the forebrain, projections to Dx originate in Dc, the ventral region of the dorsal part of the ventral telencephalon (vVd), a subsection of the central zone of the ventral telencephalon (Vc1), and the lateral zone of the ventral telencephalon (Vl). Afferent projections to Dx have also been found to originate in Dm and the dorsal Dl (dDl), yet to a smaller extent, and the preoptic area (POA). Projections from outside the forebrain were restricted to PTh, an ascending connection reminiscent of the one reported in the yellowfin goby (Hagio et al. 2021, 2018).

Extratelencephalic afferent projections to Dx have been investigated in two gobiiforms, the peacock gudgeon (Gutjahr et al. 2025) and the yellowfin goby (Hagio et al. 2021), whereas intratelencephalic connections have only been investigated in the peacock gudgeon. Recent immunohistochemical and fluorescent in situ hybridization investigations in the lizard goby (Kawaguchi et al. 2019) revealed the presence of different neuronal markers, such as substance P (SP), calretinin (calret), vglut2a, and gad65. Intriguingly, these markers were not evenly distributed across Dx‐subregions. We probed the neurochemical landscape of Dx in the peacock gudgeon by investigating the presence of different substances using immunohistochemistry against tyrosine hydroxylase (TH), parvalbumin (PV), SP, calret, neurofilament heavy chain (NFH), choline acetyltransferase (ChAT), and gamma‐aminobutyric acid (GABA). We found differential presence of these molecules within Dx and some of the nuclei projecting to Dx. Our immunohistochemical analysis complements previous tract tracing experiments delineating the ascending connectivity to Dx and suggests differential information processing within Dx.

## 2 Materials and Methods

### 2.1 Animal Maintenance

We used 26 adult peacock gudgeons (2.6 ± 0.1 cm standard body length and 0.5 ± 0.1 g body mass), which were obtained commercially (www.tropicwater.eu, Rodgau, Germany; www.petra‐aqua.com, Prague, Czech Republic) and bred in the in‐house animal facility at the University of Graz. Animals were held in groups of up to 40 individuals with an L:D‐cycle of 13:11 at 25°C. Upon arrival, commercially acquired fish were housed at the facility for at least 1 week prior to experiments.

### 2.2 Tissue Collection, Fixation, and Processing

For immunohistochemical experiments, animals were deeply anesthetized in 0.05% tricaine methanesulfonate (MS‐222, PharmaQ Ltd, Overhalla, Norway) in tank water for 15 min. Fish were measured (body mass and standard length) and decapitated. Brains were extracted in 0.1 M PB and immersion‐fixed in 4% PFA in 0.1 M PB for antibody‐specific timespans (see Table 1). After fixation, brains were transferred into 30% sucrose in 0.1 M PB overnight for cryoprotection, subsequently embedded in OCT embedding medium (CellPath Ltd., Newton, Powys, UK), and sectioned to 24–40 µm transversal sections on a cryostat (LEICA CM3000, Wetzlar, Germany or SLEE MEV, SLEE medical GmbH, Nieder‐Olm, Germany). For each antibody, we performed a secondary‐antibody‐only control following the identical procedure but leaving out the primary antibody. In these fish, brains were cut in two series (one to be incubated with and the other without primary antibody).

**TABLE 1:** Primary antibodies.

Antigen target	Raised in	Clonality	Manufacturer	Catalogue#	Lot#	Fixation time	Dilution	*N*
Tyrosine hydroxylase (TH)	Mouse	Monoclonal	Immunostar	22941	1602001	2 h	1:500	3
Substance P (SP)	Rat	Monoclonal	Novus Biologicals	NB100 65219	148697	2 h	1:50	3
Parvalbumin (parv)	Mouse	Monoclonal	Merck Millipore	P3088	n.a.	2 h	1:1000	1
Parvalbumin (parv)	Rabbit	Polyclonal	Swant	PV27	2014	2 h	1:1000	3
Calretinin (calret)	Rabbit	Polyclonal	Swant	7697	1893‐0114	o.n.	1:1000	3
Gamma‐aminobutyric acid (GABA)	Rabbit	Polyclonal	Merck Millipore	A2052	0000154656	15 min	1:100	3
Choline acetyltransferase (ChAT)	Goat	Polyclonal	Merck Millipore	AB144P	3206341	15 min	1:100	3
Neurofilament heavy chain (NFH)	Mouse	Monoclonal	DSHB	RT97	n.a.	2 h	1:500	3

### 2.3 Antibody Processing

Unless stated otherwise, all steps were performed at room temperature on an orbital shaker (Rotamax 120, Heidolph, Schwabach, Germany). Slides were first washed twice in 0.01 M (1×) PBS, followed by a permeabilization wash in 1× PBS containing 0.3% Triton TX‐100 (PBS‐T, ROTH, Karlsruhe, Germany) and subsequently blocked for 1 h with 1% bovine serum albumin (BSA, ROTH) in PBS‐T (blocking solution). Sections were incubated with primary antibodies (for details see Table 1) diluted in blocking solution for 2 h at RT and subsequently moved to 4°C overnight. The next day, sections were washed in PBS‐T three times for 10 min, incubated with diluted secondary antibodies (see Table 2 for details) in blocking solution for 2 h, and subsequently washed twice in PBS‐T for 10 min. Sections were counterstained with DAPI (3 µM in PBS‐T, MERCK Millipore, Burlington, MA, US), washed twice in 1× PBS for 5 min and twice in 0.25× PBS for 2 min. Sections were coverslipped in anti‐fade mounting medium (ROTI Mount FluorCare, ROTH; VECTASHIELD, Vector Laboratories, Newark, CA, US) and sealed with nail polish.

**TABLE 2:** Secondary antibodies.

Antigen target	Raised in	Fluorophore	Company	Incubation time (h)	Dilution
Rabbit	Donkey	Alexa‐Fluor 488	Invitrogen	2	1:500
	Donkey	Alexa‐Fluor 647	Invitrogen	2	1:500
Mouse	Donkey	Alexa‐Fluor 546	Invitrogen	2	1:500
	Donkey	Alexa‐Fluor 647	Invitrogen	2	1:500
Goat	Donkey	Alexa‐Fluor 488	Invitrogen	2	1:500
Rat	Rabbit	Alexa‐Fluor 488	Jackson ImmunoResearch	2	1:200

### 2.4 Antibody Specificity

#### 2.4.1 Tyrosine Hydroxylase

The monoclonal antibody targeting TH (Immunostar, Cat#: 22941, RRID: AB_572268) was raised in mouse using purified TH from rat PC12 cells. The antibody targets the catalytic core of TH and has previously been used in the nervous systems of several teleosts (Bryant et al. 2016; Rosner et al. 2018; Semenova et al. 2014). No cross‐reactivity has been observed with dihydropterdine reductase, dopamine‐β‐hydroxylase, phenylethanolamine‐*N*‐methyltransferase, phenylalanine hydroxylase, or tryptophan hydroxylase (Immunostar Datasheet).

#### 2.4.2 Parvalbumin

For immunohistochemistry against PV, we used a monoclonal antibody targeting PV (Merck Millipore, Cat#: P3088, RRID: AB_477329) raised in mouse using purified frog muscle PV as immunogen. PV is recognized in a Ca^2+^‐ion dependent manner in many animals, including teleosts (Holmgren and Sheets 2021; Pusch et al. 2013). No cross‐reactivity with other EF‐hand family members (including calmodulin, intestinal calcium‐binding protein, S100A2 (S100L), S100A6 (calcyclin), the a‐chain of S‐100 (i.e., in S‐100a and S‐100ao), or the b‐chain (i.e., in S‐100a and S‐100b), myosin light chain, and troponin has been reported (Merck Millipore Datasheet). Additionally, we used a polyclonal antibody targeting PV (Swant, Cat#: PV27, RRID: AB_2631173) raised in rabbit against recombinant rat PV. The antibody has been shown to target PV in mammals (Santos et al. 2016) and teleosts (De Miguel and Álvarez‐Otero 2017) and has been shown not to recognize this protein in PV knockout mice (Swant Datasheet).

#### 2.4.3 Calretinin

The polyclonal antibody against calret (Swant, Cat#: CR 7697, RRID: AB_2619710) was raised in rabbit using recombinant human calret with a 6‐His tag at the N‐terminal. The antibody specifically targets calret and has been shown to work in mammals (Medalla et al. 2023) and teleosts (Folgueira and Clarke 2024; Porter and Mueller 2020; Turner et al. 2016). No cross‐reactivity with calbindin D‐28k or other known calcium binding proteins has been reported.

#### 2.4.4 Substance P

The monoclonal antibody against SP (Novus Biologicals, Cat#: NB100 65219, RRID: AB_965531, Hybridoma clone: NC1/34, isotype: IgG2a) targets the COOH‐terminal. The antibody was raised in rat using BSA‐conjugated SP. No reactivity was observed with Leu‐ or Met‐enkephalin, somatostatin, or beta‐endorphin; however, 5% reactivity could be observed with eledoisin (Novus Biologicals Datasheet). Successful labeling of nervous system tissue has been performed in mouse (Pelayo et al. 2014) and the lizard goby (Kawaguchi et al. 2019), another gobiiform fish.

#### 2.4.5 Choline Acetyltransferase

The polyclonal antibody against ChAT (Merck Millipore, Cat#: AB144P, RRID: AB_2079751) was raised in goat using human placental enzyme. This antibody targets ChAT in cholinergic neurons in, for example, mice (Jeong et al. 2015) and teleosts (Giraldez‐Perez et al. 2009; Mueller et al. 2004; Rosner et al. 2018).

#### 2.4.6 Neurofilament Heavy Chain

The monoclonal antibody against NFH (DSHB, Cat#: RT97, RRID: AB_528399, Hybridoma Clone: RT97, isotype: MIgG1) was raised in mouse against rat semi‐purified neurofilaments from brain homogenate. The antibody has been shown to react with NFH in teleosts (Velasco et al. 2000) nervous system tissue and is known to cross‐react with neurofilament medium.

#### 2.4.7 Gamma‐Aminobutyric Acid

The polyclonal antibody against GABA (Merck Millipore, Cat#: A2052, RRID: AB_477652) was raised in rabbit using BSA‐conjugated GABA as immunogen. The antibody has been shown to react to GABA and GABA‐KLH but not to BSA. It was also shown to target GABA in several species, including teleosts (Mueller et al. 2006; Tripp and Bass 2020; Turner et al. 2022).

### 2.5 Imaging and Analysis

Stained sections were imaged on an epifluorescence microscope (ZEISS Axio Imager 2, ZEISS, Jena, Germany). Selected slices were additionally imaged with a confocal microscope (SP8, Leica, Wetzlar, Germany). Image post‐processing was performed in ImageJ (FIJI distribution, (Schindelin et al. 2012)), during which we only adjusted brightness and contrast as necessary. All image processing was performed uniformly across the whole image. For a comparison between test and control experiments, we imaged non‐primary antibody experiments and adjusted their brightness and contrast with identical parameters as used for sections processed with primary antibodies. For visualization of the immunoreactivity patterns of individual subregions, we categorized intensity of immunoreactivity from weakest to strongest through visual analysis and plotted our results using a custom‐written Python script building on Seaborn (Waskom 2021). Figures were generated using Paint.Net (version 5.0.3, dptPDN LLC, Seattle, WA, US) and CorelDraw (version 21.3.0.755, Corel Corporation, Ottawa, Canada).

## 3 Results

Labeling of structures in the yellowfin goby Dx has previously shown dense fiber fields of small projections that were barely discriminable due to their “sponge‐like” fiber density (Hagio et al. 2018, injections into PTh). After performing immunohistochemistry on brain sections of the peacock gudgeon, we observed similar dense fiber field labeling after immunostaining with some of our antibodies.

### 3.1 Tyrosine Hydroxylase

**FIGURE 1:**
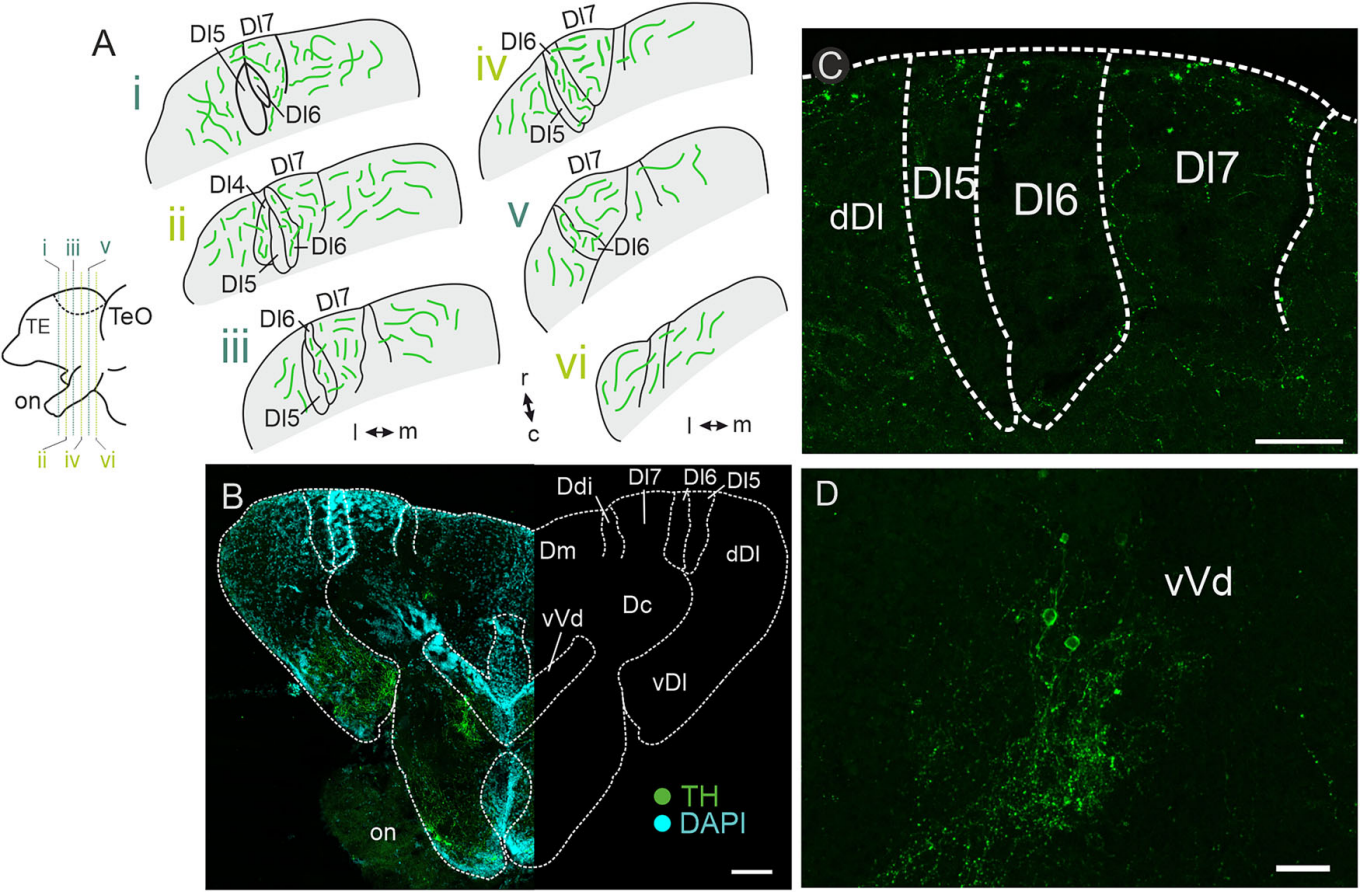
**Immunoreactivity against tyrosine hydroxylase (TH)**. (A) Schematic overview of TH‐immunoreactive fibers distributed along the rostro–caudal axis of Dx. Bottom left: side view of schematized forebrain. (B) Overview image of an exemplary section stained against TH (left). The right side of the image displays a schematic overview for orientation. (C) Distribution of TH‐immunoreactive fibers throughout Dx. Note the absence of fibers within Dl5. (D) TH‐immunoreactive neurons within vVd of the ventral telencephalon. Colors: green = TH‐immunoreactive structures, cyan = DAPI. Scale bars: B = 100 µm; C = 50 µm; D = 20 µm. Abbreviations can be found in the list of abbreviations.

Immunohistochemical staining against TH revealed sparse fibers evenly distributed along the rostro–caudal axis of Dl7, Dl6, and Dl4 (Figure 1A), but almost none of these fibers could be found in Dl5. We found no TH‐immunoreactive neurons within either of the soma‐dense or the fiber‐rich Dx‐subregions (Figure 1B,C), indicating that the TH‐immunoreactive fibers must originate from neurons located outside of Dx. No labeling was present in our secondary‐only controls (Figure S1A). Our staining revealed that TH‐immunoreactive somata within the telencephalon were abundant in vVd of the ventral telencephalon (Figure 1D).

### 3.2 Parvalbumin

**FIGURE 2:**
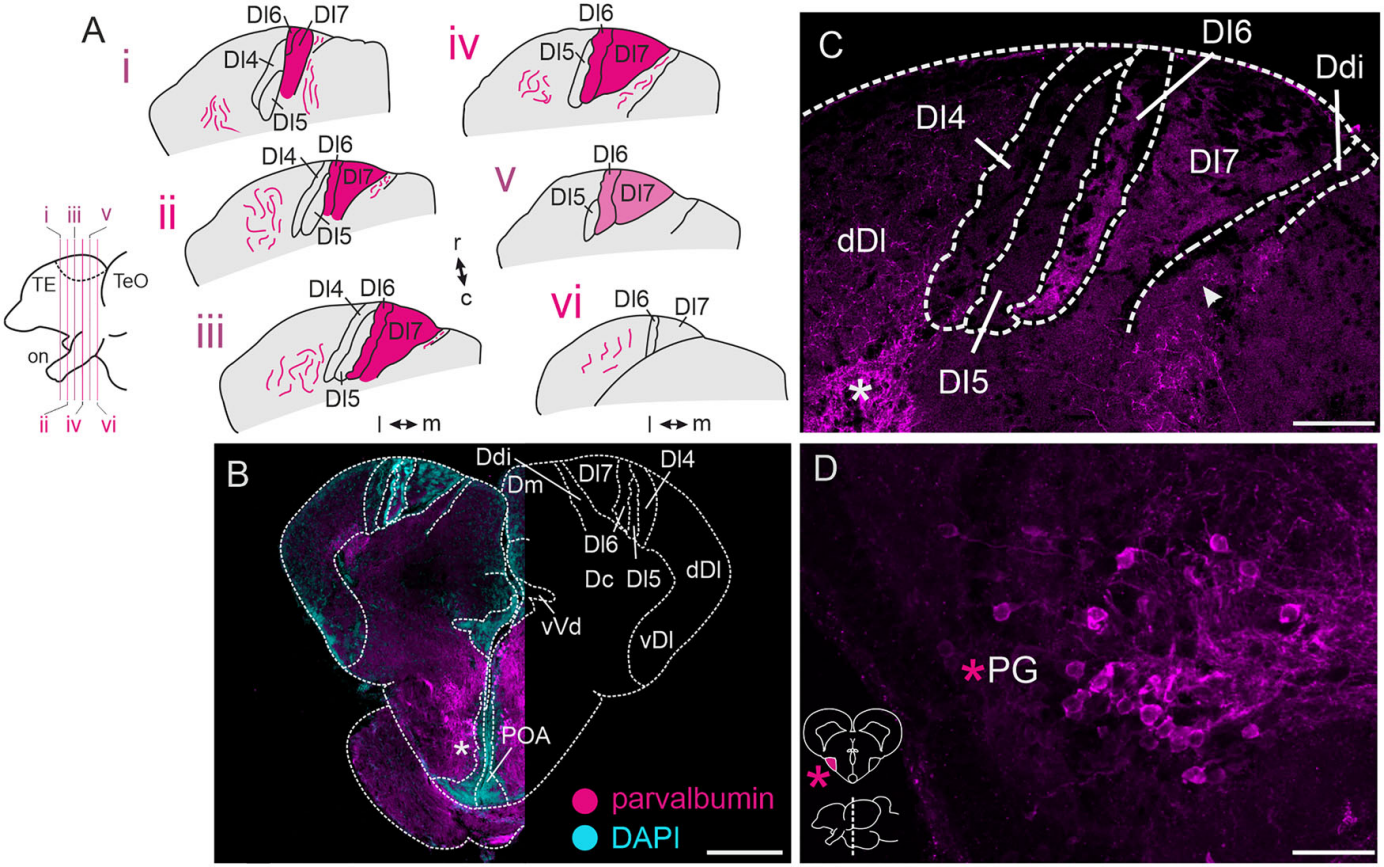
**Immunoreactivity against parvalbumin (PV)**. (A) Schematic overview of parvalbumin‐immunoreactive fibers across the rostro–caudal axis of Dl4–Dl7 within the dorsal telencephalon. Bottom left: side view of schematized forebrain. Dark magenta areas indicate dense projection fields; lighter magenta indicates less dense projection fields. (B) Overview image of one section stained against parvalbumin (left). The right side of the image shows a schematized section for orientation. (C) Higher magnification of dense immunoreactive putative terminal fields within Dl6 and Dl7. Note the extremely weak labeling of these dense fields within Dl5 and Dl4. (D) Parvalbumin‐immunoreactive neurons within PG. Inset shows the rostro–caudal position of the section within the brain (bottom) and an overview of the location of PG (top). Scale bars: B = 200 µm; C = 50 µm; D = 25 µm. Colors: magenta = parvalbumin‐immunoreactive structures, cyan = DAPI. Abbreviations can be found in the list of abbreviations.

Immunohistochemistry against PV revealed densely labeled, putative terminal fields within Dl6 and Dl7, which were observable from the rostral to the caudal end of Dx (Figure 2A), whereas Dl4 and Dl5 were devoid of this intense labeling. Observed fibers were sparser within more caudal sections of Dx (Figure 2A; for overview, see Figure 2B). Within Dx, we could not find any PV‐immunoreactive somata (Figure 2C), indicating putative terminal fields, with somata being located in a different brain region. PV‐immunoreactive neurons were, however, found in the POA (Figure 2B, asterisk) and some parts of vVd (not shown). At more caudal levels, we found numerous PV‐immunoreactive neurons within PG (Figure 2D). Adjacent to Dx, we identified additional immunoreactive fibers within Ddi (Figure 2C, arrowhead) and dDl (Figure 2C, asterisk). The control experiment (no‐primary) did not reveal any labeling (Figure S1B).

### 3.3 Substance P

**FIGURE 3:**
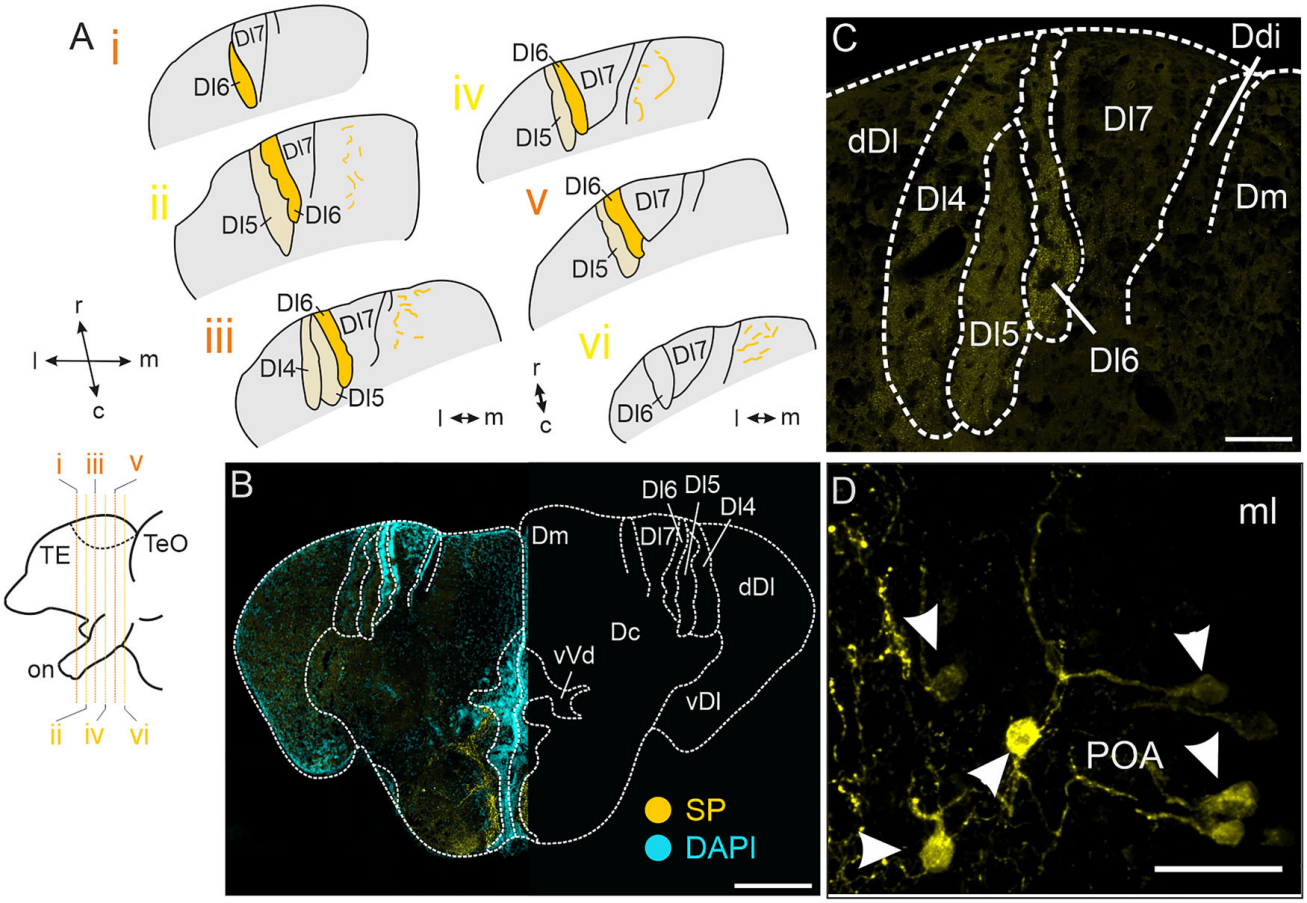
**Immunoreactivity against substance P (SP**). (A) Schematic overview of substance P‐immunoreactive fibers across the rostro–caudal axis of Dx. Darker yellow areas are dense projection fields; lighter yellow areas are less dense projection fields. Bottom left: side view of schematized forebrain. (B) Overview image of one section stained against substance P (left). The right side of the image displays a schematized section for orientation. (C) Higher magnification of dense immunoreactive putative terminal fields within Dl4, Dl5, and particularly Dl6. Note the absence of labeling within Dl7. (D) Substance P‐immunoreactive neurons within the POA, marked by arrowheads. Scale bars: B = 200 µm; C = 50 µm; D = 25 µm. Colors: yellow = substance‐P‐ir structures, cyan = DAPI. Abbreviations can be found in the list of abbreviations.

We found an even distribution of SP‐immunoreactive putative terminal fields through the rostro–caudal axis of Dx (Figure 3A). The densest labeling was found within Dl6 (Figure 3B,C), whereas Dl7 was devoid of SP staining. We found SP‐immunoreactive somata in the POA (Figure 3D, arrowheads) and sparsely also in vVd (not shown). As our immunohistochemical experiments did not reveal SP‐immunoreactive somata within Dx, we concluded that the labeled putative terminal fields belong to somata located in a different brain region. Our no‐primary control did not show any labeled fibers or somata (Figure S1C).

### 3.4 Calretinin

**FIGURE 4:**
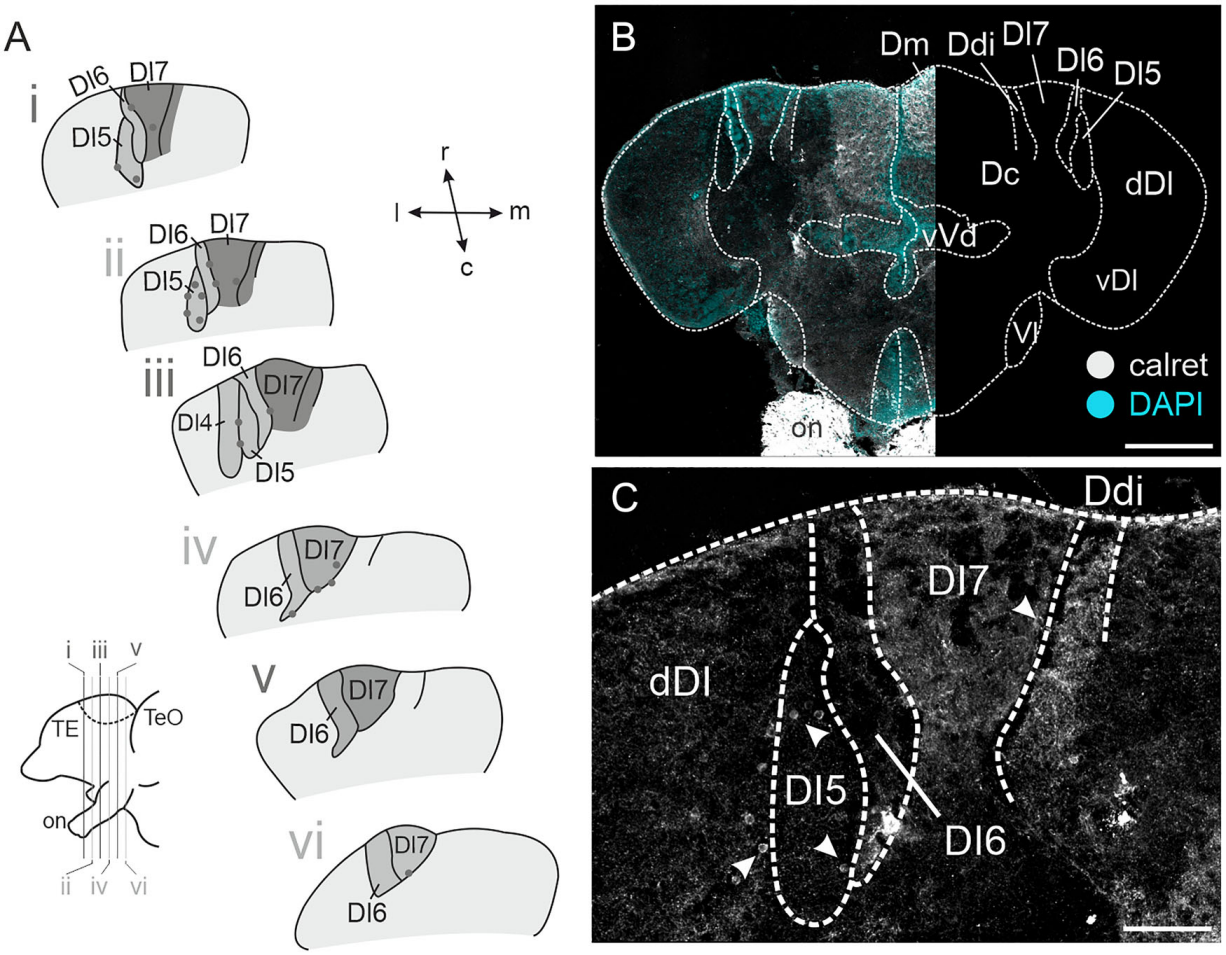
**Immunoreactivity against calretinin (calret)**. (A) Schematic overview of calretinin‐ir fiber fields across the rostro–caudal axis of Dx. Dark gray areas are dense fiber fields; lighter gray areas are less dense fiber fields. Calretinin‐ir neurons are portrayed as small, dark‐gray circles. Bottom left: side view of schematized forebrain. (B) Overview image of a section stained against calretinin (left). The right side of the image displays an overview of a schematized section for orientation. (C) Higher magnification of immunoreactive fiber fields within Dx. Arrowheads point toward calretinin‐immunoreactive neurons. Scale bars: B = 200 µm, C = 50 µm. Colors: gray = calretinin‐immunoreactive structures, cyan = DAPI. Abbreviations can be found in the list of abbreviations.

Within Dx, immunohistochemical stainings revealed immunoreactive fibers and putative terminal fields, which were observed throughout the rostro–caudal extent of Dx (Figure 4A), with the densest terminal fields being located in Dl7 and the neighboring Ddi (Figure 4B,C). We found dense calret‐immunoreactive terminal fields within Dm (Figure 4B); a thick fiber tract could be observed leading up to Dm from the ventral side, putatively arising from the forebrain bundles (not shown). Further caudally, we found a population of calret‐immunoreactive neurons located within the rostral PG and dense putative terminal fields within an area just dorsal of PG (not shown). Our no‐primary control experiment revealed no immunoreactive fibers (Figure S1D). Additionally, sparsely distributed calret‐immunoreactive somata could be observed throughout Dl4–Dl7 (Figure 4C, arrowheads).

### 3.5 Neurofilament Heavy Chain

**FIGURE 5:**
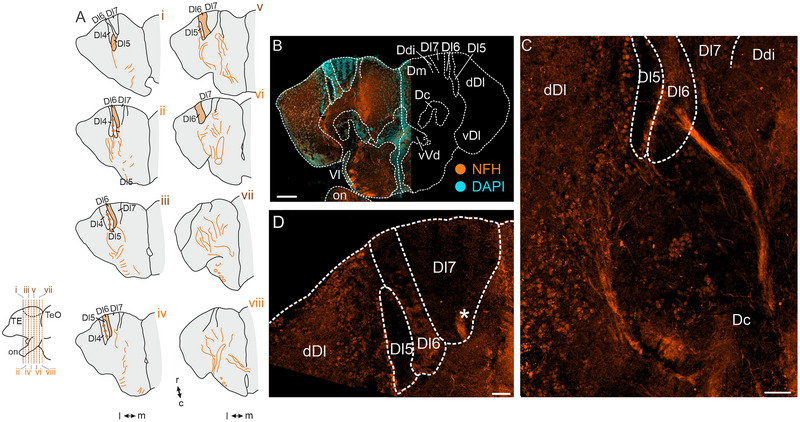
**Immunoreactivity against neurofilament heavy chain**. (A) Schematic overview of NFH‐immunoreactive fibers and fields across the rostro–caudal axis of Dx. Orange areas indicate dense NFH‐immunoreactive fiber fields. Bottom left: side view of schematized forebrain. (B) Overview image of a section stained against neurofilament heavy chain (NFH, left). The right side of the image displays a schematized section for orientation. (C) Higher magnification of an NFH‐immunoreactive fiber tract reaching Dl6. Dense NFH‐immunoreactive fibers present within Dl6, as well as Dc. (D) Distribution of NFH‐immunoreactive fiber fields within Dx. Scale bars: B = 100 µm, C,D = 25 µm. Colors: orange = NFH‐immunoreactive structures, cyan = DAPI. Orange circles indicate NFH‐immunoreactive somata within the lateral border of Dl5, adjacent to dDl. Abbreviations can be found in the list of abbreviations.

Immunohistochemistry against NFH revealed dense labeling of Dl6 (Figure 5A–D), alongside many NFH‐immunoreactive somata in, for example, Vl and vVd (not shown). At rostral levels of Dx, we could find a tract that was connected to Dl6, which seemed to be projecting from the ventral telencephalic area and perhaps arises from the forebrain bundles (Figure 5Ai–iii,C: detail). More caudally, we also found a tract that entered Dl7 (Figure 5Aiv,D, asterisk). Within Dx, we also found NFH‐immunoreactive somata within the lateral border of Dl5, adjacent to dDl, which showed numerous NFH‐ir somata (Figure 5C,D). Our no‐primary control experiment revealed no immunoreactive structures (Figure S1E).

### 3.6 Choline Acetyltransferase

**FIGURE 6:**
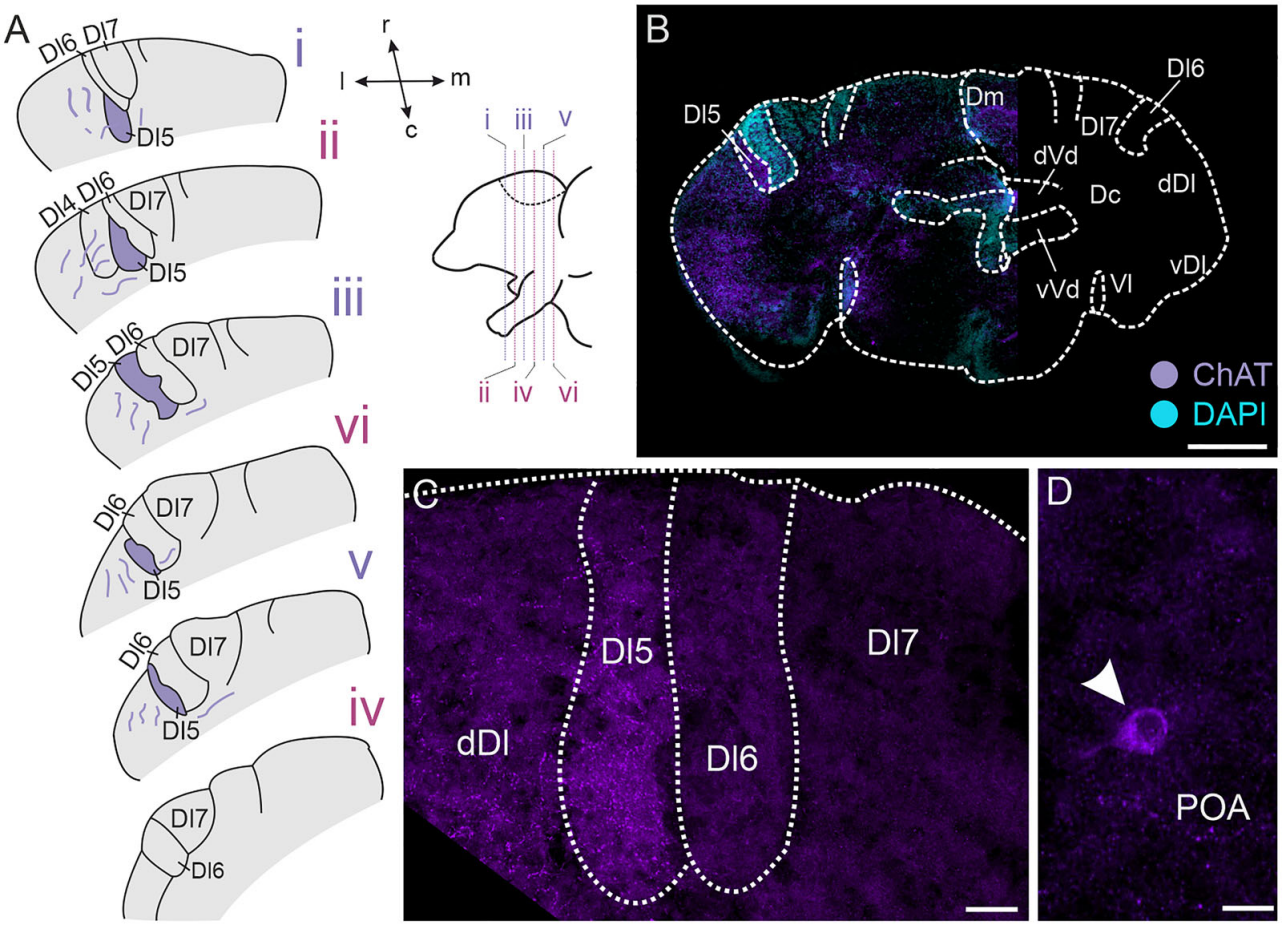
**Immunoreactivity against choline acetyl transferase (ChAT)**. (A) Schematic overview of choline acetyltransferase (ChAT)‐immunoreactive fiber fields across the rostro–caudal axis of Dx. Purple areas are dense fiber fields. Right: side view of schematized forebrain. (B) Overview image of a section stained against ChAT (left). The ride side of the image displays a schematized overview of a section for orientation. (C) Higher magnification of Dx showing immunoreactive fiber field within Dl5. (D) ChAT‐immunoreactive soma within the POA, indicated by white arrowhead. Scale bars: B = 200 µm, C = 25 µm, D = 10 µm. Colors: purple = ChAT‐immunoreactive structures, cyan = DAPI. Abbreviations can be found in the list of abbreviations.

Immunohistochemistry against ChAT revealed a dense putative ChAT‐immunoreactive projection field confined to Dl5 at all rostro–caudal levels (Figure 6A). Other Dx‐subregions displayed weaker ChAT‐immunoreactivity (Figure 6B,C); however, no ChAT‐ir somata were labeled within Dx. Additionally, we found fibers adjacent to Dx, located within dDl (Figure 6A–C). A few ChAT‐immunoreactive neurons could be found in the POA (Figure 6D, arrowhead). No‐primary control experiments revealed no immunoreactive structures (Figure S1F).

### 3.7 Gamma‐Aminobutyric Acid

**FIGURE 7:**
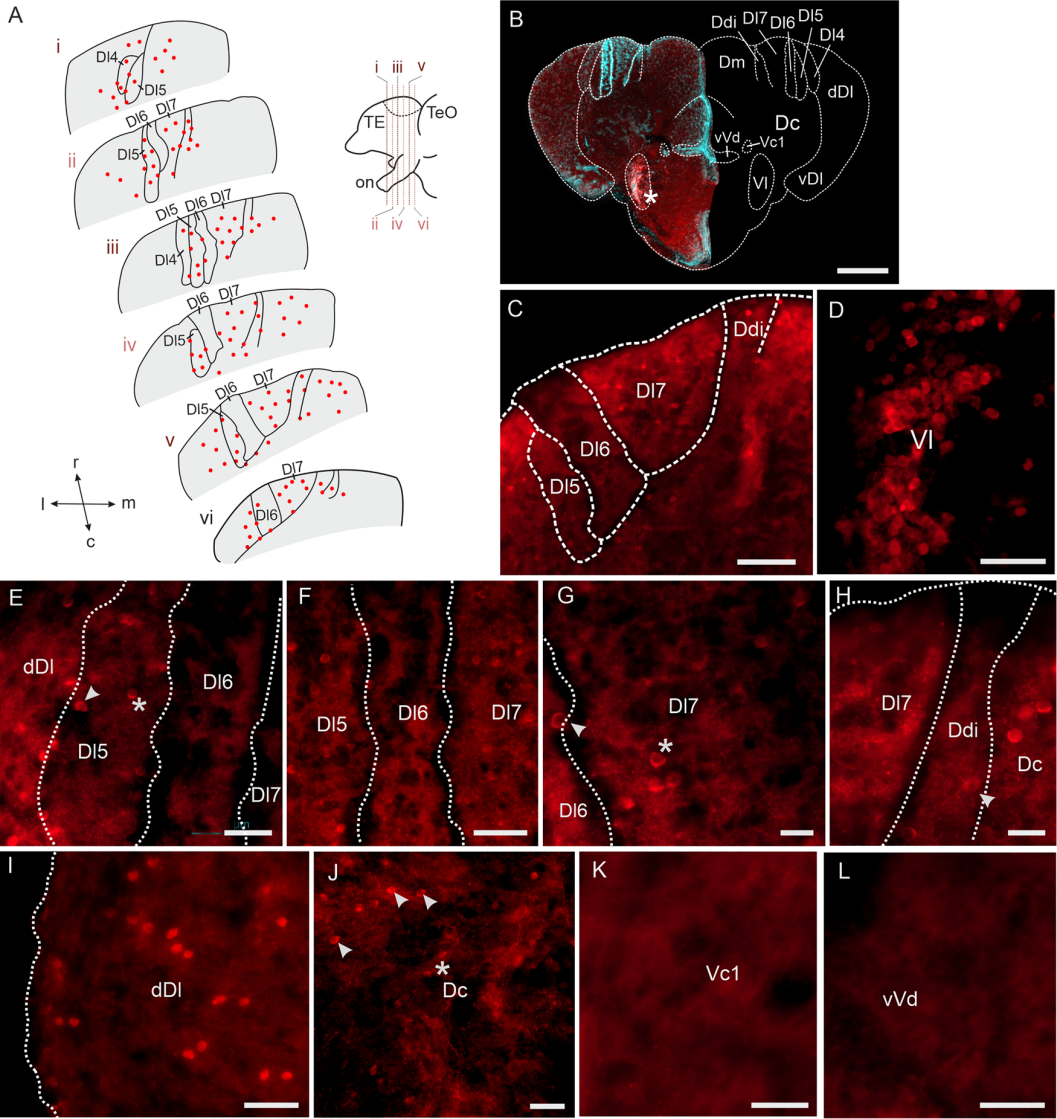
**Immunoreactivity against gamma‐aminobutyric acid (GABA)**. (A) Schematic overview of GABA‐immunoreactive neurons across the rostro–caudal axis of Dx. Filled circles depict the presence of GABA‐immunoreactive somata within an area. Top right: side view of schematized forebrain. (B) Overview image of a section stained against GABA (left). The right side of the image displays a schematized section for orientation. (C) GABA‐immunoreactive somata within Dx. (D) Higher magnification of immunoreactive somata within Vl. (E) Higher magnification image of more lateral subregions within Dx: GABA‐immunoreactive somata are present in Dl5 (asterisk) and the soma‐dense layer lateral to Dl5, bordering dDl (arrowhead). (F) GABA‐immunoreactive somata within central Dx: Dl6 is almost devoid of GABA‐immunoreactive somata. (G) GABA‐immunoreactive somata within Dl7 (asterisk) and the soma‐dense layer lateral to Dl7, separating Dl7 and Dl6 (arrowhead). (H) GABA‐immunoreactive soma in Ddi (arrowhead). (I) GABA‐immunoreactive somata within dDl. (J) GABA‐immunoreactive soma in Dc (asterisk) and GABA‐ir somata in an area in dorsal Dc (arrowhead), where neurons are more loosely distributed. (K) Vc1 without any GABA‐immunoreactive somata. (L) vVd without GABA‐immunoreactive neurons. Colors: red = GABA‐ir structures, cyan = DAPI. Scale bars: B = 200 µm; C = 50 µm; D,I = 25 µm; E,F,J,K,L = 20 µm; G,H = 10 µm. Abbreviations can be found in the list of abbreviations.

Immunohistochemical staining against GABA revealed numerous immunoreactive somata throughout the rostro–caudal extent of Dx, with almost no immunoreactive somata located within Dl6 (Figure 7A,B (overview)). GABA‐immunoreactive neurons in Dx were sparse and almost regularly distributed throughout Dl5 and Dl7 (Figure 7C). Compared to the densely clustered GABA‐immunoreactive neurons in Vl (Figure 7D). Our non‐primary control did not reveal any immunoreactive structures (Figure S1G).

Next, we looked at the presence of neurons within individual Dx subregions. Throughout Dx, we found GABA‐immunoreactive somata located in the soma‐dense layer lateral to the Dl5 subregion (Figure 7E, arrowhead) and within Dl5 (asterisk). In contrast, almost no immunoreactive somata were located within Dl6 and the soma‐dense layers surrounding it (Figure 7F,G). Immunoreactive somata were found within Dl7 (Figure 7G, asterisk) and the neighboring Ddi (Figure 7H, arrowhead).

Outside Dx, but within the telencephalon, we found many GABA‐immunoreactive somata within, for example, Dc (Figure 7H,J asterisk), dDl (Figure 7I), and an area located in between Dc and Dx (Figure 7J, arrowheads). Additional cell bodies were visible in the POA (Figure 7B) and a particular hotspot of GABA‐immunoreactive somata within Vl (Figure 7D). Vc1 (Figure 7K) and vVd (Figure 7L) were mostly devoid of GABA‐immunoreactive somata.

## 4 Discussion

**FIGURE 8:**
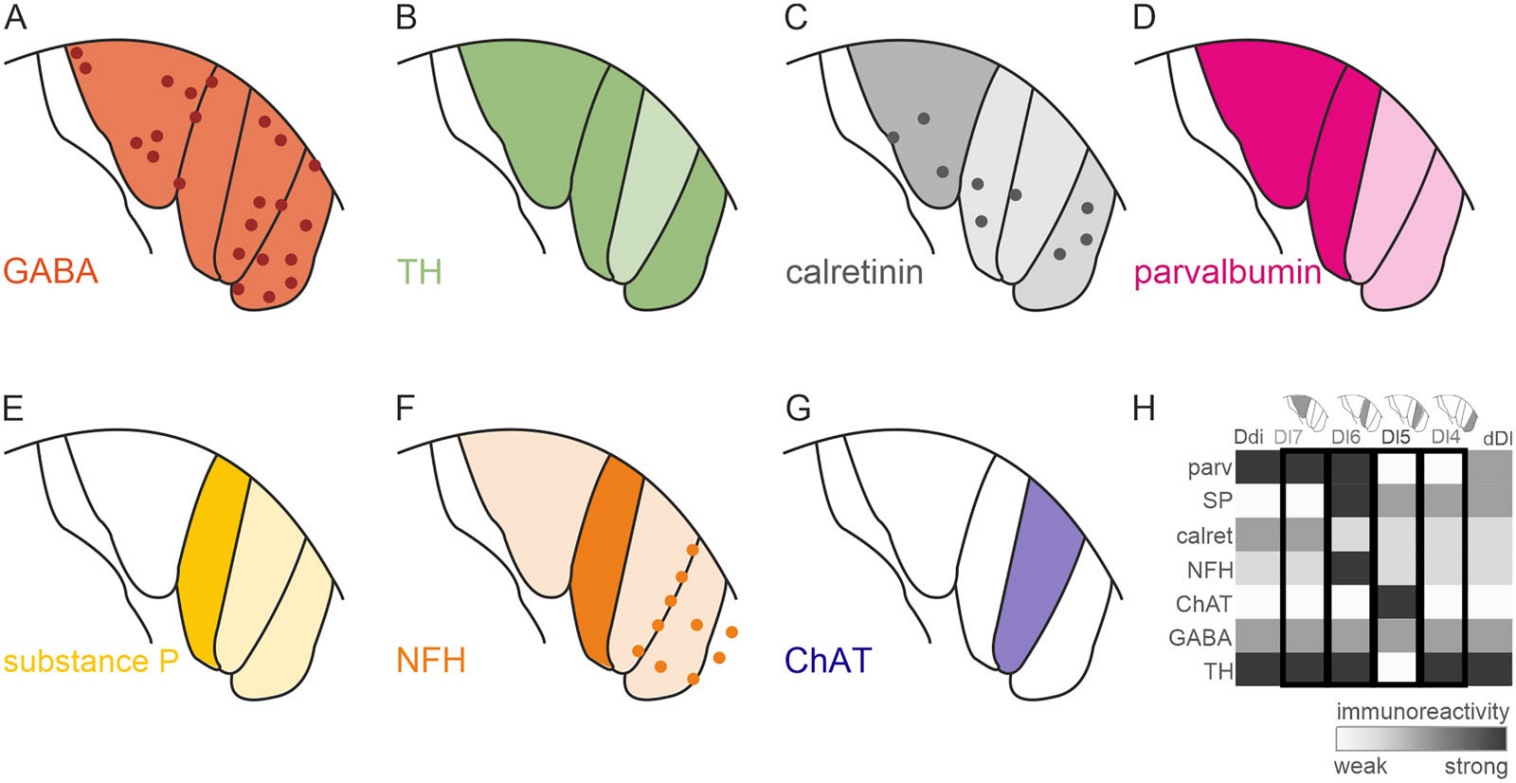
**Summary of different antigen immunoreactive structures within Dx**. Schematic drawings of Dx with color coded immunoreactivity profiles of GABA (A), tyrosine hydroxylase (TH) (B), calretinin (C), parvalbumin (D), substance P (E), neurofilament heavy chain (NFH) (F), and choline acetylcholinesterase (ChAT) (G). (H) Summary of the immunohistochemical profile of Dl4–Dl7 and adjacent regions of Ddi and dDl. Abbreviations can be found in the list of abbreviations. GABA, gamma‐aminobutyric acid; TH, tyrosine hydroxylase.

We found that each Dx‐subregion had their own distinctive immunohistochemical profile: Dl7 contained GABA‐ir fibers and somata (Figure 8A), TH‐ir fibers (Figure 8B), calret‐ir fiber fields, sparse calret‐ir somata (Figure 8C), dense putative parv‐ir putative terminal fields (Figure 8D), no SP‐ir structures (Figure 8E), NFH‐ir fibers (Figure 8F), and almost no ChAT‐ir structures (Figure 8G). In Dl6, we found GABA‐ir fibers and almost no GABA‐ir somata (Figure 8A), TH‐ir fibers (Figure 8B), calret fiber fields, and sparse calret‐ir somata (Figure 8C), parv‐ir fiber fields (Figure 8D), dense SP‐ir fiber fields (Figure 8E), dense NFH‐ir fiber fields, and almost no ChAT‐ir structures (Figure 8G). Within Dl5, there were GABA‐ir fibers and GABA‐ir somata (Figure 8A), only extremely few TH‐ir fibers (Figure 8B), calret fiber fields and sparse calret‐ir somata (Figure 8C). We also found lightly parv‐ir putative terminal fields (Fig. 8D), lightly SP‐ir fiber fields (Figure 8E), very little NFH‐immunoreactivity and ChAT‐ir dense putative terminal fields (Figure 8G). Dl4 was characterized by GABA‐ir fibers and somata (Figure 8A), TH‐ir fibers (Figure 8B), calret‐ir fiber fields and sparse calret‐ir somata (Figure 8C), lightly parv‐ir putative terminal fields (Figure 8D), almost no SP‐immunoreactivity (Figure 8E), sparse NFH‐ir fibers, as well as NFH‐ir somata (Figure 8F), and almost no ChAT‐ir structures (Figure 8G). Although each Dx‐subregion had their own neurochemical signature, the immunoreactivity of Dl4 most resembled the one of dDl, and the Dl7‐immunoreactivity most resembled the one of Ddi (Figure 8H). This differentiation of subregions according to different immunoreactivity indicates differential innervation of individual subregions in Dx.

### 4.1 Tyrosine Hydroxylase

TH is an enzyme involved in the catalysis of catecholamines, such as dopamine, norepinephrine, and epinephrine. As we performed immunohistochemical experiments for TH, we cannot distinguish between dopaminergic, noradrenergic, and adrenergic immunoreactivity and hence use the term *catecholaminergic*. Within Dx, we found very sparse labeled fibers in Dl4, Dl6, and Dl7, whereas Dl5 was mostly devoid of TH‐immunoreactive fibers. As these fibers were small and very sparse, we could only detect them at high magnifications. As no TH‐positive somata were found within Dx, we conclude that these fibers are from neurons projecting to Dx. As the only extratelencephalic source of ascending input to Dx, that is, PG, did not reveal any TH‐immunoreactive neurons, we propose that the catecholaminergic input to Dx is intratelencephalic. Within the remaining telencephalon, most of the TH‐immunoreactive neurons were located ventro‐medially within both hemispheres: TH‐immunoreactive somata could be detected within Vv and vVd. As vVd has been identified as a source of ascending input to Dx (Gutjahr et al. 2025), we propose that the input from vVd to Dx is (at least in part) catecholaminergic.

Interestingly, our results differ from those obtained in the lizard goby, *R. flumineus* (Kawaguchi et al. 2019), and the great blue spotted mudskipper, *Boleophthalmus pectinirostris*: For these two oxudercids, TH‐fibers could not be detected within Dx. In other teleosts that lack a Dx, sparse TH‐immunoreactive fibers were observed within the dorsal (Dd) and/or dorsolateral (dDl) region of the telencephalon in, for example, *Apteronotus leptorhynchus* (dDl, Dd, (Sas et al. 1990)), the plainfin midshipman, *Porichthys notatus* (dDl, (Forlano et al. 2014; Goebrecht et al. 2014)), the Burton's haplo, *Astotilapia burtoni* (dDl, (O'Connell et al. 2011)), the Senegalese sole, *Solea senegalensis* (Dd, dDl (Rodríguez‐Gómez et al. 2000)), and the European seabass, *Dicentrarchus labrax* (dDl, Dd (Batten et al. 1993)). All of these regions share a similar location with the gobiiform Dx.

Although intense immunoreactivity of fibers and somata within medial subregions of the ventral telencephalon (including vVd) is found in several teleosts (Anadón et al. 2002; Batten et al. 1993; Borgonovo et al. 2021; Forlano et al. 2014; Goebrecht et al. 2014; Hornby et al. 1987; Kaslin and Panula 2001; Meek and Joosten 1993; O'Connell et al. 2011; Parafati et al. 2009; Rink and Wullimann 2001; Roberta et al. 2023; Rodríguez‐Gómez et al. 2000; Sas et al. 1990; Vetillard et al. 2002), we currently do not know if these structures are homologous to those found in the peacock gudgeon. Their similar location (often not easily comparable), together with their TH‐immunoreactivity, however, suggests strong similarities.

### 4.2 Parvalbumin

We found very dense PV‐ir putative terminal fields in Dl7 and Dl6. As we could not find PV‐ir neurons within Dx, we concluded that these fibers must originate from outside of Dx. Based on previous tract tracing studies (Gutjahr et al. 2025) and our immunoreactivity results, two candidate nuclei appear likely: vVd and the preglomerular complex (PG), as both project to Dx and showed PV‐ir somata. There is a remarkable similarity in staining patterns between injections into the PG region (Gutjahr et al. 2025) and PV‐immunohistochemistry in Dx, where intensely labeled dense terminal fields are present in Dl6 and Dl7, but not Dl5. Similar PV‐immunoreactive afferent connectivity patterns have been reported in the zebrafish forebrain, where PV‐immunoreactive input to the pallium could also be traced back to the PG and the Vv/Vd area (Mueller et al. 2011).

To our knowledge, there are no previous reports for PV‐immunoreactivity in gobiiform forebrains, but PV immunohistochemical experiments conducted in other teleosts have investigated the distribution of PV across teleostean forebrains: Although PV‐ir neurons were not observed in the peacock gudgeon pallium, pallial PV‐ir somata were found sparsely distributed in the pallium in the Masu salmon, *Oncorhynchus masou* (Puschina and Varaksin 2011), the tench, *Tinca tinca* (Briñón et al. 1994; Crespo et al. 1999), and the zebrafish, *Danio rerio* (Dl, (Mueller et al. 2011)). Similar to the peacock gudgeon, PV‐ir fiber systems could be observed in parts of the pallium and medial zone of subpallium (including areas where vVd is located gobies) in the tench and zebrafish (Crespo et al. 1999; Mueller et al. 2011; Porter and Mueller 2020). Whether these structures are homologous remains to be investigated.

### 4.3 Substance P

Within Dx, SP‐ir dense putative terminal fields were seen predominantly in Dl6, whereas Dl5 and Dl4 showed weaker labeling and were completely devoid of labeled structures. As there were no SP‐ir neurons within Dx, we concluded that the SP‐immunoreactivity in Dx must originate from outside of Dx. SP‐ir somata could be observed sparsely in vVd and the POA. The vVd is known to project to Dx, and sparse projections have also been shown for the POA to Dx. Therefore, we suggest that SP‐input to Dx must come from either or both these two regions.

In contrast to the peacock gudgeon, the lizard goby shows a different immunoreactivity pattern: SP‐ir fibers were observed in Dl7, Dl6, and Dl4, not however in Dl5 (Kawaguchi et al. 2019). SP‐immunoreactive fibers were also shown in the vVd and Vc1. Stainings against an SP‐precursor, protachykinin, have revealed immunoreactive somata in Vc1, which could be the potential source of afferent SP connections to Dx. Future immunohistochemical experiments targeting protachykinin in the peacock gudgeon might reveal additional potential sources of SP‐connections to Dx.

With the exception of *Carassius auratus*, where SP‐immunoreactive somata have been reported in Dp (Sharma et al. 1989), to our knowledge, no other SP‐immunoreactive somata have been reported. Throughout studies, SP‐immunoreactivity within the telencephalon was reported as sparse, with fibers found in areas of the medio‐ventral TE (in similar locations as the peacock gudgeon vVd) in the rainbow trout, *Salmo gairdneri* (Vecino et al. 1989), the sea bass, *D. labrax* (Moons et al. 1992), the Atlantic salmon, *Salmo salar* (Holmqvist and Ekström 1991), and in *A. leptorhynchus* (Dulka et al. 1995; Weld and Maler 1992). SP‐immunoreactive fibers were also found in these subregions of the ventral telencephalon, which supports our finding of fibers within vVd. Only one study reported SP‐immunoreactive neurons within the POA (*D. labrax* (Moons et al. 1992)).

### 4.4 Calretinin

Within Dx, we found calret‐ir somata and fiber fields, with denser fiber fields located in Dl7 and the adjacent Ddi. Within the remaining dorsal forebrain, calret‐immunoreactive somata were distributed very sparsely and intermingled with sparse calret‐immunoreactive fibers found in dDl. Within Dm, however, we found extremely dense calret‐immunoreactive fiber fields.

Among gobies, calret‐immunoreactive structures have been reported for the lizard goby (Kawaguchi et al. 2019), in which calret positive fibers could be observed within Dl7, Dl6, and Dl4. Calret‐immunoreactive somata could be found in Vl, vVd, Dc, and Dl4. In the peacock gudgeon, sparse immunoreactive somata were found throughout Dl4–7 with the majority located in Dl4.

Comparing our findings to those of other teleosts, we find similarities but also some differences regarding the calret‐ir: Sparse calret‐ir fibers and scarce somata have been reported in both Dd and dDl in a number of teleosts (Castro et al. 2003, 2006; Deshpande and Jadhao 2015; Díaz‐Regueira and Anadón 2000). The extremely dense Dm‐calret‐immunoreactive terminal field could also be found (Castro et al. 2003, 2006; Díaz‐Regueira and Anadón 2000). Numerous calret‐ir somata have been reported in the ventral telencephalic area, close to the ventricle in, for example, *D. rerio* (Castro et al. 2006), *Salmo trutta* Fario (Castro et al. 2003), and *Eigenmannia virescens* (Wong 1997). In our immunohistochemical experiments, we could not find such populations of calret‐immunoreactive neurons located in or close to vVd or dVd, which would be the anatomically corresponding areas in the peacock gudgeon.

### 4.5 Neurofilament Heavy Chain

We performed immunohistochemistry against NFH to better understand the ascending fiber system to Dx. We found that the largest fiber tract reaches Dl6 and either just innervates the most ventral area, from which intrinsic connectivity could spread information throughout Dl6, or splits into fine fibers, whose final destinations were impossible to resolve. Caudal to this Dl6‐associated tract, there is another tract that connects to the ventral region of Dl7. We could not determine, whether both these tracts fuse to enter the lateral forebrain bundle or enter Dc or Vl. We found that some somata were immunoreactive, particularly within Dl5. We were not able to identify if the label was present due to immunoreactivity of the soma, or if the signal originated from axons innervating those neurons in a calyx‐like manner.

### 4.6 Choline Acetyltransferase

Within Dx, we found a ChAT‐immunoreactive putative terminal field only within Dl5. As we could not identify any ChAT‐immunoreactive somata within Dx, we concluded that the cholinergic input to Dl5 must originate from regions outside of Dx. During recent tract tracing experiments, we have revealed the major ascending connections to Dx (see Section 1), and the only source of afferent connections that we identified as ChAT‐immunoreactive in this study was limited to few very sparsely labeled neurons within the POA. We thus hypothesize that the POA provides the cholinergic input to Dx.

To our knowledge, ChAT immunoreactivity has not yet been investigated in the forebrain of gobies. However, comparisons with other teleosts revealed that sparse fibers could be found within the pallium of several teleosts (Brantley and Bass 1988; Castro et al. 2003; Clemente et al. 2004; Ekström 1987; Giraldez‐Perez et al. 2009; Mueller et al. 2004; Pérez et al. 2000). ChAT‐ir somata were absent from the pallium in all of these fishes, indicating extrapallial, cholinergic input. Generally, ChAT‐ir neurons were extremely sparse in the forebrain and diencephalon and were confined to the POA, where ChAT‐immunoreactive somata have also been reported (Casini et al. 2018; Castro et al. 2003; Clemente et al. 2005; Giraldez‐Perez et al. 2009; Kaslin et al. 2004; Mueller et al. 2004; Pérez et al. 2000; Toscano‐Márquez et al. 2021).

### 4.7 Gamma‐Aminobutyric Acid

GABA‐immunoreactive somata and fibers could both be observed within Dx; therefore, we do not know which of the fibers in Dx correspond to Dx‐intrinsic GABAergic neurons and which to projecting neurons with somata located outside Dx, if any. However, we found many GABA‐immunoreactive neurons within Vl, an area that projects to Dx in the peacock gudgeon (Gutjahr et al. 2025), which indicates that Dx could receive GABAergic input from Vl.

As in the peacock gudgeon, sparsely distributed GABArgic somata were also seen in the pallium of *Poecilia sphenops* (Hotha and Ganesh 2023), *O. masou* (Puschina and Varaksin 2011), *C. auratus* (Martinoli et al. 1990), *Anguilla anguilla* (Médina et al. 1994), and gymnotiforms (Giassi et al. 2012). With exception of gymnotiforms, all of the above studies showed many GABA‐ir somata in the medial ventral telencephalon (including areas corresponding to our vVd), which was not the case for the medial ventral telencephalon (vVd, Vc1) in the peacock gudegon.

## 5 Conclusion

The differential immunoreactivity of the Dx subregions indicates that the individual subregions receive different or graded input from other regions of the brain: Dl7 receives PV‐ and TH‐immunoreactive input likely from PG and vVd. Dl6 receives SP‐immunoreactive input, likely from the POA, and PV‐ir input, likely from PG. Dl5 receives ChAT‐immunoreactive input, likely from the POA, but no PV‐immunoreactive input from PG. None of the Dx subregions receive the exact same pattern of immunoreactivity, which is intriguing, as this suggests that inputs are differentially processed within Dl7–Dl4. Although the function of Dx remains to be investigated, this study provides evidence for different functional compartments across Dx.

## Author Contributions

Ruth Gutjahr and Boris P. Chagnaud conceptualized the study. Boris P. Chagnaud and Ruth Gutjahr developed the methodology. Ruth Gutjahr and Maximilian S. Bothe performed all experiments and analyses. Ruth Gutjahr, Maximilian S. Bothe, and Boris P. Chagnaud interpreted the data. Ruth Gutjahr and Maximilian S. Bothe visualized the data. Ruth Gutjahr wrote the original draft. Ruth Gutjahr, Maximilian S. Bothe, and Boris P. Chagnaud reviewed and edited the manuscript. Boris P. Chagnaud supervised the study and provided funding.

## Peer Review

The peer review history for this article is available at https://publons.com/publon/10.1002/cne.70097.

## Supporting information




**Supplementary Material**: cne70097‐sup‐0001‐SuppMat.docx

**Supplementary Figure**: cne70097‐sup‐0002‐FigureS1.png

## Data Availability

The data that support the findings of this study are available from the corresponding author upon reasonable request.
