# Supplementary material for: Immunohistochemical Profile of a Conspicuously Organized Structure in the Dorsal Forebrain of the Peacock Gudgeon, Tateurndina ocellicauda
Source: J Comp Neurol. 2025 Oct 15;533(10):e70097. doi: 10.1002/cne.70097 (PMC12528548; doi:10.1002/cne.70097)
Supplement: Supplementary file 1 — Supplementary Material: cne70097‐sup‐0001‐SuppMat.docx [file CNE-533-e70097-s002.docx]

**Figure S1 | Secondary-only controls of IHC experiments.** Imaging of “test” and “control” slices. Immunohistochemical development was performed identical with the exception of using 1x PBST + blocking buffer without primary antibody on control slides during primary incubation. Imaging was performed using identical exposure time and brightness for test and control conditions, respectively. Brightness/Contrast adjustments were performed on images for visualization with the same values for test and control images. Left panels show test, right panels show control images. Primary antibodies: (A) mouse-anti-tyrosine hydroxylase. (B) rabbit-anti-parvalbumin. (C) rat-anti-substance P. (D) rabbit-anti-calretinin. (E) mouse-anti-neurofilament-heavy-chain. (F) goat-anti-choline acetyltransferase. (G) rabbit-anti-gamma-aminobutyric acid. Scale bars: all 50 µm.
