# Supplementary figures and images for: Immunohistochemical Profile of a Conspicuously Organized Structure in the Dorsal Forebrain of the Peacock Gudgeon, Tateurndina ocellicauda
Source: J Comp Neurol. 2025 Oct 15;533(10):e70097. doi: 10.1002/cne.70097 (PMC12528548; doi:10.1002/cne.70097)

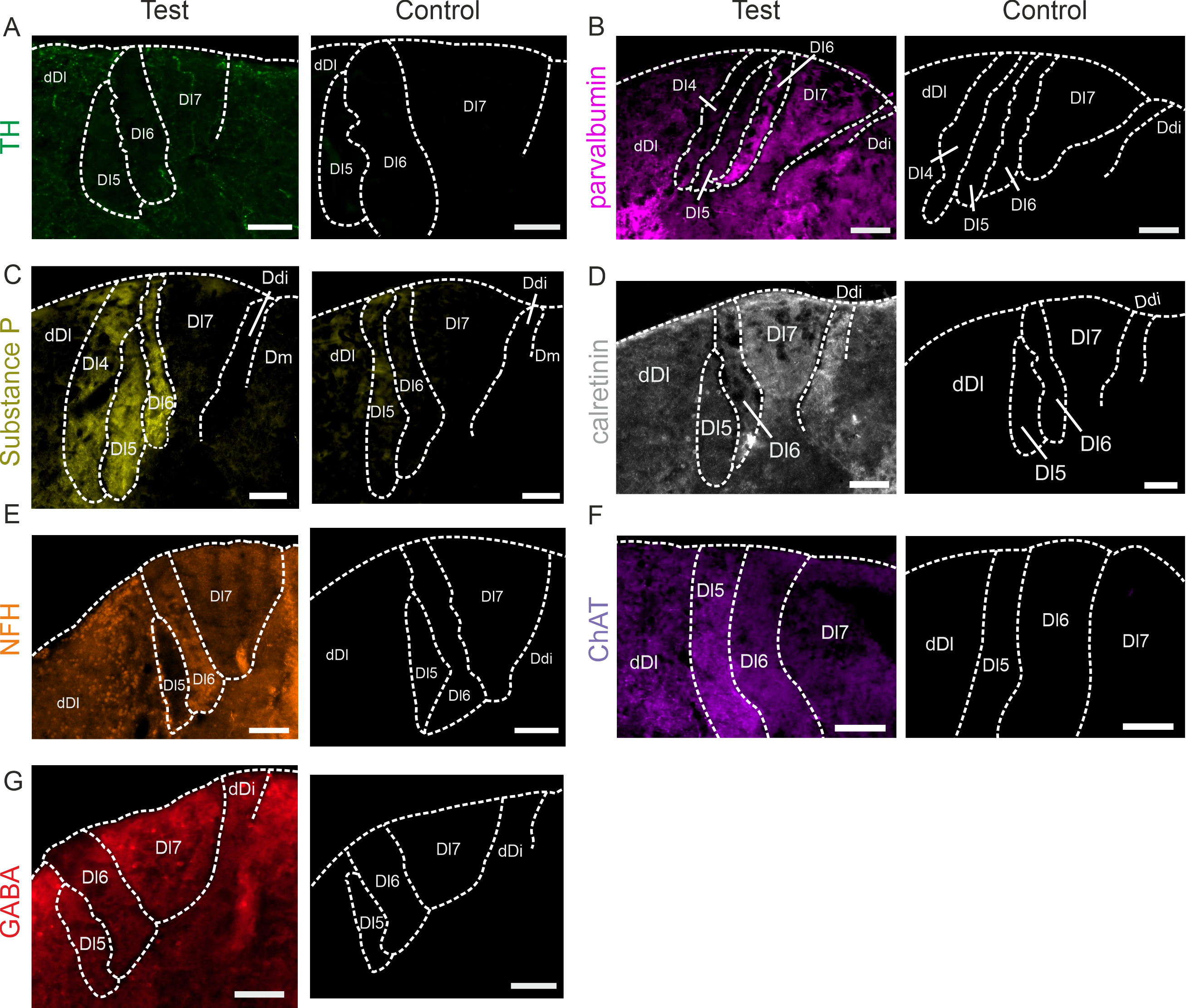

Supplement: Supplementary file 2 — Supplementary Figure: cne70097‐sup‐0002‐FigureS1.png [file CNE-533-e70097-s001.png]
